# GPER signalling in both cancer-associated fibroblasts and breast cancer cells mediates a feedforward IL1β/IL1R1 response

**DOI:** 10.1038/srep24354

**Published:** 2016-04-13

**Authors:** Paola De Marco, Rosamaria Lappano, Ernestina Marianna De Francesco, Francesca Cirillo, Marco Pupo, Silvia Avino, Adele Vivacqua, Sergio Abonante, Didier Picard, Marcello Maggiolini

**Affiliations:** 1 Department of Pharmacy and Health and Nutritional Sciences, University of Calabria, 87036 Rende, Italy; 2 Breast Cancer Unit, Regional Hospital, 87100 Cosenza, Italy; 3 Department of Cell Biology, Faculty of Sciences, and Institute of Genetics and Genomics of Geneva, University of Geneva, Geneva Switzerland

## Abstract

Cancer-associated fibroblasts (CAFs) contribute to the malignant aggressiveness through secreted factors like IL1β, which may drive pro-tumorigenic inflammatory phenotypes mainly acting via the cognate receptor named IL1R1. Here, we demonstrate that signalling mediated by the G protein estrogen receptor (GPER) triggers IL1β and IL1R1 expression in CAFs and breast cancer cells, respectively. Thereby, ligand-activation of GPER generates a feedforward loop coupling IL1β induction by CAFs to IL1R1 expression by cancer cells, promoting the up-regulation of IL1β/IL1R1 target genes such as PTGES, COX2, RAGE and ABCG2. This regulatory interaction between the two cell types induces migration and invasive features in breast cancer cells including fibroblastoid cytoarchitecture and F-actin reorganization. A better understanding of the mechanisms involved in the regulation of pro-inflammatory cytokines by GPER-integrated estrogen signals may be useful to target these stroma-cancer interactions.

Cancer-associated fibroblasts (CAFs) as main players within the tumor microenvironment contribute to the growth, expansion and dissemination of cancer cells^1^. For instance, CAFs generate a dynamic signalling network through the secretion of several factors that stimulate adjacent malignant cells toward tumor progression^2^. In addition, CAFs may drive a worse cancer phenotype mostly via a paracrine action exerted by growth factors and chemokines released in the tumor microenvironment^2^,^3^. Increasing evidence have also assessed that CAFs act as mediators of neoplastic-promoting inflammation due to their production of pro-inflammatory cytokines^1^,^4^,^5^. The interleukin 1 (IL-1) family of cytokines plays an important role in diverse pathophysiological conditions, including the malignant disease^6^. In particular, IL1α and IL1β and the cognate receptors namely IL1R1 and IL1R2, are expressed in numerous types of cancer cells^7^,^8^. Accordingly, IL1α and IL1β knockout mice exhibited impaired skills to develop tumors and angiogenesis^9^,^10^. Likewise, the interleukin-1 receptor antagonist, named IL-1Ra, decreased the inflammatory response and inhibited tumor progression in mice^11^. High levels of IL1β within the tumor microenvironment have been associated with increased recurrence and metastasis in breast cancer^4^,^9^,^12^,^13^. In this regard, it has been shown that breast cancer cells exposed to IL1β may acquire an invasive phenotype through diverse structural changes as the loss of cell-cell contact, the acquisition of a fibroblastoid cytoarchitecture and cell scattering^14^,^15^. Moreover, a positive correlation between IL1β levels and estrogens was found in breast tissue biopsies and the ability of estrogens to stimulate IL1β production was recently reported both *in vitro* and in breast cancer xenografts^10^,^11^.

Estrogens stimulate breast cancer progression mainly by binding to and activating the estrogen receptor (ER)α and ERβ, which regulate the expression of genes involved in the proliferation, migration and survival of tumor cells^16^. The G protein estrogen receptor (GPR30/GPER) can also mediates the action of estrogens in both normal and malignant cell contexts^17^,^18^. Ligand-activated GPER induces a network of signal transduction pathways including epidermal growth factor receptor (EGFR), intracellular cyclic AMP, calcium mobilization, MAPK and PI3K^19^. In addition, GPER mediates a specific gene signature associated with cell growth, migration and angiogenesis in estrogen-sensitive tumors^20^,^21^,^22^,^23^,^24^. The potential of GPER in mediating stimulatory effects has been also evidenced in CAFs derived from patients with breast cancer, suggesting that the action of GPER may involve a functional interaction between these components of the tumor microenvironment and cancer cells^20^,^25^,^26^. The role of GPER has been highlighted even in the cardiovascular, neurological and immunological systems as well as in the inflammatory state^27^,^28^. For instance, in knockout mice GPER was shown to be required for thymic atrophy and thymocyte apoptosis induced by estrogens and the selective GPER agonist G-1^29^. Moreover, estrogenic GPER signalling stimulated the invasion and migration of breast cancer cells through IL8-activated CXC receptor-1 (CXCR1)^30^. In endometrial cancer cells, GPER triggered the secretion of IL6, a pleiotropic cytokine that has been associated with both inflammation and cancer^31^.

Here, we show that ligand-activated GPER triggers the EGFR/ERK/PKC signal transduction pathway generating a feedforward loop that couples IL1β induction by CAFs to IL1R1 expression by cancer cells. Our findings highlight the potential of GPER in contributing to the functional interplay between cancer cells and the surrounding stroma toward biological responses that drive the progression of breast cancer.

## Results

### GPER mediates induction of IL1β expression by E2 and G-1 in CAFs

**Figure 1:**
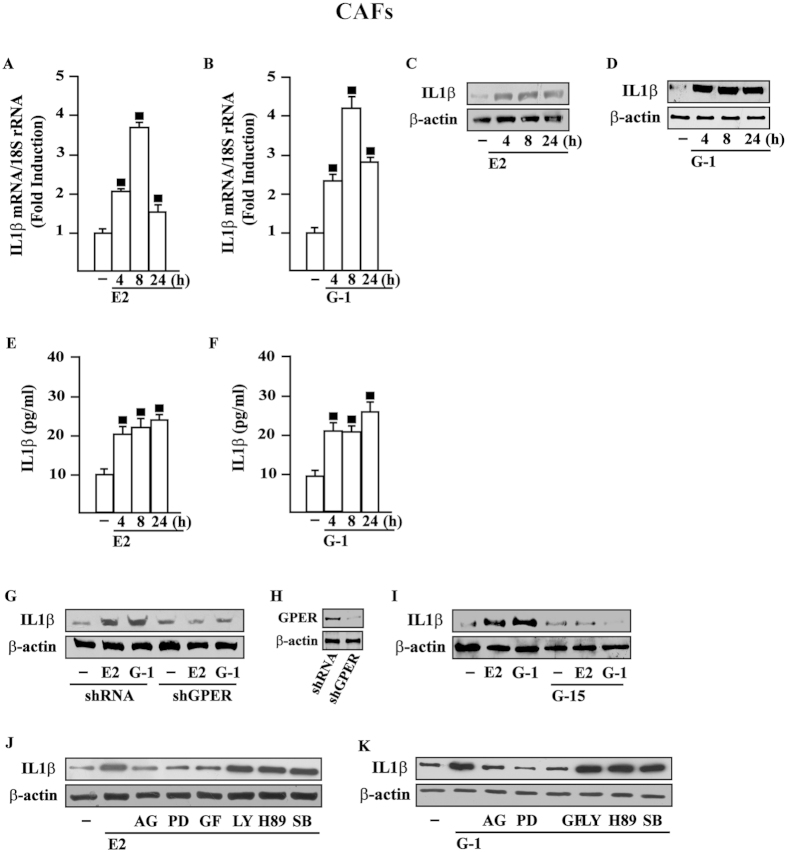
GPER mediates the up-regulation of IL1β expression by E2 and G-1 in CAFs. 10 nM E2 (**A**) and 100 nM G-1 (**B**) induce IL1β mRNA expression, as evaluated by real-time PCR. Data obtained in three independent experiments performed in triplicate were normalized to 18S expression and shown as fold changes of IL1β expression upon E2 and G-1 treatments respect to cells exposed to vehicle (−). (◼) p < 0.05 for cells receiving treatments versus vehicle. 10 nM E2 (**C**) and 100 nM G-1 (D) up-regulate IL1β protein expression, as indicated. (**E**,**F**) ELISA of IL-1β in supernatants collected from E2 or G-1 treated CAFs. Data are representative of 5 independent experiments. (**G**) The up-regulation of IL1β protein levels induced by 10 nM E2 and 100 nM G-1 is abrogated in CAFs transfected for 24 h with shGPER and then treated for 8 h with vehicle (−), 10 nM E2 and 100 nM G-1. (**H**) Efficacy of GPER silencing. (**I**) The induction of IL1β protein expression observed upon treatments for 8 h with 10 nM E2 or 100 nM G-1 is abolished using 100 nM GPER antagonist G-15. (**J**,**K**) IL1β protein levels in CAFs treated for 8 h with vehicle (−), 10 nM E2 and 100 nM G-1 alone or in combination with 1 μM EGFR inhibitor AG1478 (AG), 1 μM MEK inhibitor PD98059 (PD), 1 μM PKC inhibitor GF109203X (GF), 1 μM PI3K inhibitor LY294,002 (LY), 1 μM PKA inhibitor H89 and 1 μM p38 MAPK inhibitor SB 203580 (SB). β-actin serves as a loading control. Results shown are representative of at least two independent experiments.

Previous studies have shown that the pro-inflammatory cytokine IL1β is regulated by estrogens in breast tissue and tumor xenografts, however the mechanisms involved remain to be elucidated^10^,^11^. In order to provide mechanistic insights into the IL1β response to estrogens within the tumor microenvironment, we began our study determining that IL1β is one of the most induced genes by ligand-activated GPER, as assessed in a nanostring analysis performed in CAFs (data not shown). In accordance with the aforementioned findings, we ascertained that E2 and G-1 induce IL1β expression in CAFs at both mRNA (Fig. 1A,B) and protein levels (Fig. 1C,D). Conversely, E2 and G-1 did not trigger IL1β stimulation in fibroblasts derived from noncancerous breast tissue (data not shown). As expected, E2 and G-1 stimulated the secretion of IL1β in CAFs medium, as determined by ELISA (Fig. 1E,F). Moreover, we established that IL1β protein induction upon E2 and G-1 exposure is no longer evident silencing GPER (Fig. 1G,H) or using the GPER antagonist G-15 (Fig. 1I). As agonist-stimulated GPER triggers the activation of diverse signal transduction pathways^19^, we then assessed that the up-regulation of IL1β triggered by E2 and G-1 is prevented in the presence of EGFR tyrosine kinase inhibitor AG, MEK inhibitor PD and PKC inhibitor GF, but not using the PI3K inhibitor LY, the PKA inhibitor H89 and the p38 MAPK inhibitor SB (Fig. 1J,K). Overall, these data indicate that E2 and G-1 induce IL1β expression through GPER-mediated signalling in CAFs.

### IL1R1 expression is regulated by E2 and G-1 through GPER in breast cancer cells

**Figure 2:**
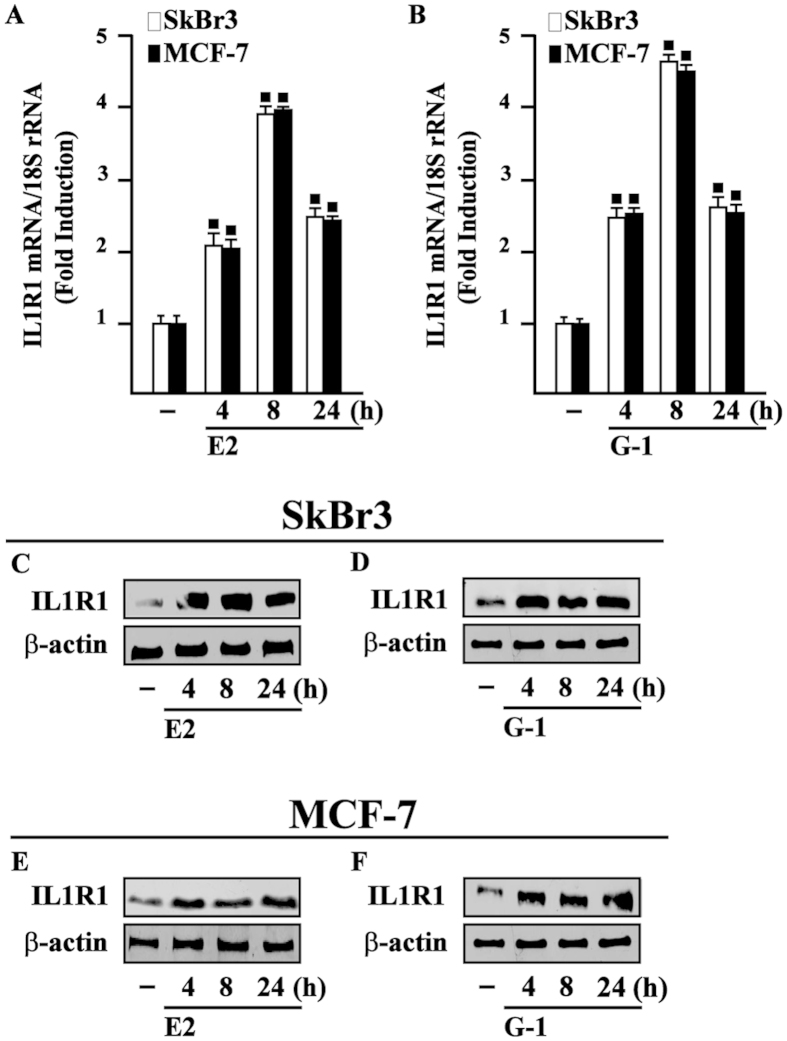
E2 and G-1 induce IL1R1 expression in SkBr3 and MCF-7 breast cancer cells. 10 nM E2 (**A**) and 100 nM G-1 (**B**) induce the mRNA expression of IL1R1, as evaluated by real-time PCR. Data obtained in three independent experiments performed in triplicate were normalized to 18S expression and shown as fold changes of IL1R1 expression upon E2 and G-1 treatments respect to cells exposed to vehicle (−). (◼) p < 0.05 for cells receiving treatments versus vehicle. Evaluation of IL1R1 protein expression in SkBr3 (**C**,**D**) and MCF-7 cells (**E**,**F**) treated with 10 nM E2 and 100 nM G-1, as indicated. β-actin serves as a loading control. Results shown are representative of at least two independent experiments.

**Figure 3:**
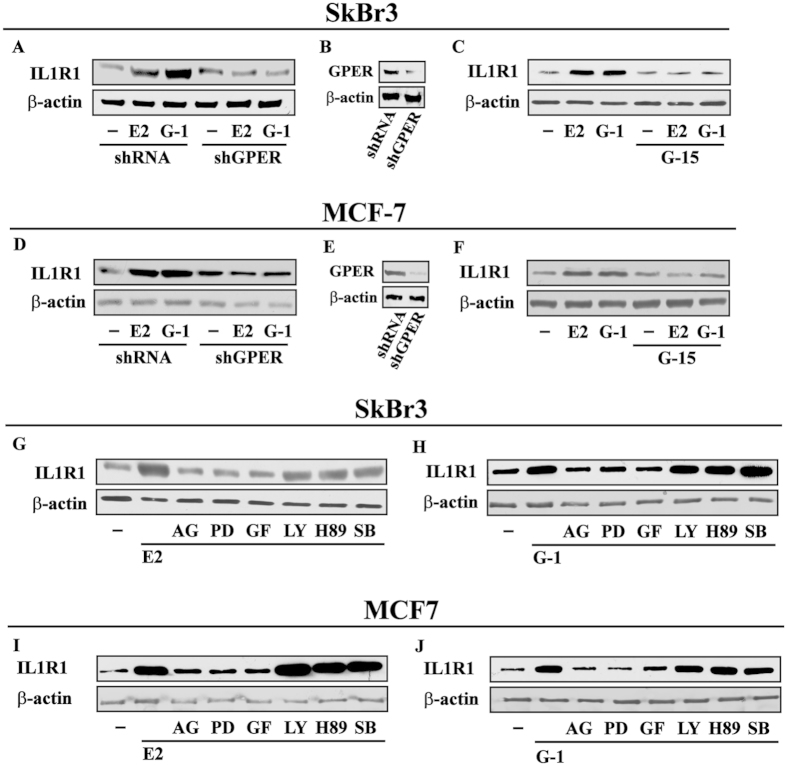
GPER mediates the up-regulation of IL1R1 expression by E2 and G-1 in SkBr3 and MCF-7 breast cancer cells. (**A**) The up-regulation of IL1R1 protein levels upon treatment for 8 h with 10 nM E2 and 100 nM G-1 is abrogated transfecting SkBr3 cells for 24 h with shGPER. (**B**) Efficacy of GPER silencing. (**C**) The induction of IL1R1 protein expression observed treating SkBr3 cells for 8 h with 10 nM E2 and 100 nM G-1 is abolished in the presence of 100 nM GPER antagonist G-15. (**D**) The up-regulation of IL1R1 protein levels upon treatment for 8 h with 10 nM E2 and 100 nM G-1 is abrogated transfecting MCF-7 cells for 24 h with shGPER. (**E**) Efficacy of GPER silencing. (**F**) The induction of IL1R1 protein expression observed treating MCF-7 cells for 8 h with 10 nM E2 and 100 nM G-1 is abolished in the presence of 100 nM GPER antagonist G-15. IL1R1 protein levels in SkBr3 cells treated for 8 h with 10 nM E2 (**G**) and 100 nM G-1 (**H**) alone or in combination with 1 μM EGFR inhibitor AG1478 (AG), 1 μM MEK inhibitor PD98059 (PD), 1 μM PKC inhibitor GF109203X (GF), 1  μM PI3K inhibitor LY294,002 (LY), 1 μM PKA inhibitor H89 and 1 μM p38 MAPK inhibitor SB 203580 (SB). IL1R1 protein levels in MCF-7 cells treated for 8 h with 10 nM E2 (**I**) and 100 nM G-1 (**J**) alone or in combination with 1 μM EGFR inhibitor AG, 1 μM MEK inhibitor PD, 1 μM PKC inhibitor GF, 1 μM PI3K inhibitor LY, 1 μM PKA inhibitor H89 and 1 μM p38 MAPK inhibitor SB. β-actin serves as a loading control. Results shown are representative of at least two independent experiments.

Pro-inflammatory factors secreted within the breast tumor microenvironment mainly act via cognate receptors expressed by cancer cells^32^. On the basis of the abovementioned results and previous studies showing that estrogens may regulate the levels of IL1R1^33^, we evaluated whether GPER mediates IL1R1 expression in breast tumor cells. As shown in Fig. 2, E2 and G-1 up-regulated the mRNA (Fig. 2A,B) and protein expression (Fig. 2C–F) of IL1R1 in both SkBr3 and MCF-7 cells. Moreover, IL1R1 protein induction by E2 and G-1 was abolished knocking-down the expression of GPER as well as in the presence of the GPER antagonist G-15 in SkBr3 and MCF-7 cells (Fig. 3A–F). Next, the up-regulation of IL1R1 by E2 and G-1 was prevented using the EGFR inhibitor AG, the MEK inhibitor PD and the PKC inhibitor GF, while the inhibitors of PI3K, PKA and p38 transduction pathways namely LY, H89 and SB, respectively, did not show any effect (Fig. 3G–J) as observed using also the ER antagonist ICI (Supplementary Fig. 1). Altogether, these results suggest that E2 and G-1 trigger the up-regulation of IL1R1 in breast cancer cells through GPER-mediated signalling.

### GPER and IL1R1 are involved in the induction of PTGES expression by E2 and G-1 in breast cancer cells

**Figure 4:**
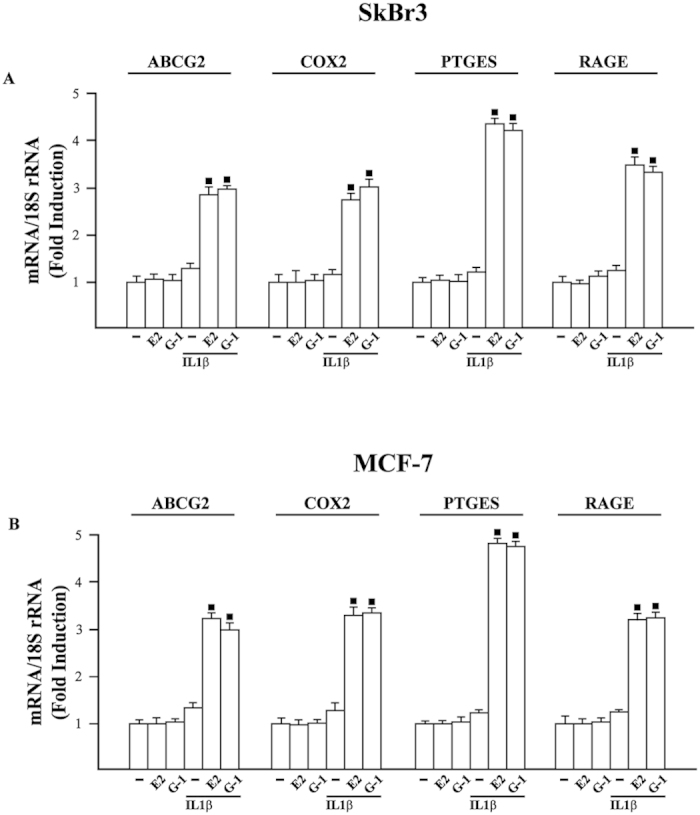
mRNA expression of ABCG2, COX2, PTGES and RAGE evaluated by real-time PCR in SkBr3 (**A**) and MCF-7 (**B**) cells treated for 8 h with vehicle (−), 10 nM E2, 100 nM G-1 and 10 ng/ml IL1β. Cells were also treated for 8 h with 10 nM E2 and 100 nM G-1 before the treatment for 8 h with 10 ng/ml IL1β, as indicated. Results obtained from three independent experiments performed in triplicate were normalized for 18S expression and shown as fold change of RNA expression respect to cells treated with vehicle. (◼) p < 0.05 for cells receiving treatments versus vehicle.

**Figure 5:**
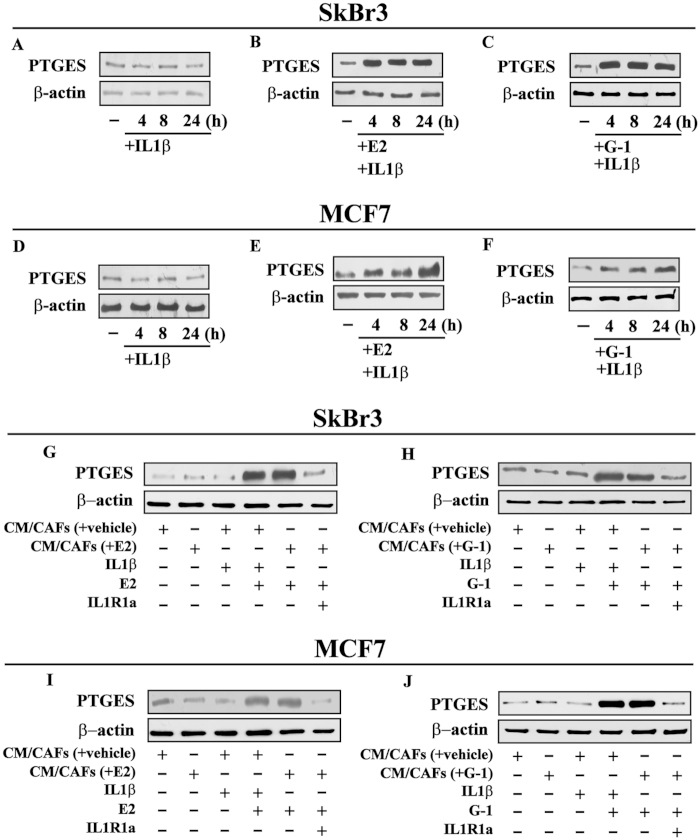
PTGES protein expression in SkBr3 (**A**–**C**) and MCF-7 (**D**–**F**) cells treated with 10 ng/ml IL1β alone or treated for 8 h with 10 nM E2 or 100 nM G-1 and then exposed to 10 ng/ml IL1β, as indicated. Protein levels of PTGES in SkBr3 (**G**,**H**) and MCF-7 (**I**–**J**) cells treated for 8 h with 10 nM E2 or 100 nM G-1 and then switched for additional 8 h to medium without serum in the presence of 10 ng/ml IL1β or conditioned medium collected from CAFs (CM/CAFs) treated for 8 h with vehicle [CM/CAFs (+vehicle)], 10 nM E2 [CM/CAFs (+E2)] and 100 nM G-1 [CM/CAFs (+G-1)]. SkBr3 and MCF-7 cells treated for 8 h with 10 nM E2 or 100 nM G-1 were also exposed to [CM/CAFs (+E2)] and [CM/CAFs (+G-1)] alone or in combination with 1 μM IL1R1 antagonist namely IL1R1a. β-actin serves as a loading control. Results shown are representative of at least two independent experiments.

In order to evaluate the transcriptional responses mediated by GPER through the up-regulation of IL1R1 in SkBr3 and MCF-7 cells, we assessed the changes of certain IL1β target genes^34^,^35^. For instance, the mRNA expression of ATP-binding cassette G2 (ABCG2), cyclooxygenase-2 (COX2), prostaglandin E synthase-1 (PTGES) and receptor for advanced glycation end products (RAGE) was stimulated only in SkBr3 and MCF-7 cells treated with E2 and G-1 before IL1β exposure (Fig. 4A,B). In accordance with these findings, we determined that the protein levels of PTGES are up-regulated by IL1β only upon E2 and G-1 exposure in SkBr3 and MCF-7 cells (Fig. 5A–F), suggesting that the increase of IL1R1 by agonist-activated GPER does contribute to the aforementioned responses. Considering that E2 and G-1 trigger the expression of IL1β in CAFs (shown in Fig. 1) and IL1R1 in breast cancer cells (shown in Fig. 2), we then assessed that conditioned medium from CAFs exposed to E2 and G-1 does induce PTGES protein expression in SkBr3 (Fig. 5G–H) and MCF-7 (Fig. 5I,J) cells exposed to E2 or G-1. Using the IL1R1 antagonist, namely IL1R1a, the up-regulation of PTGES observed in the aforementioned experimental conditions was no longer evident (Fig. 5G–J). Moreover, an increased expression of PTGES was observed treating with IL1β both SkBr3 and MCF-7 cells exposed to E2 and G-1 (Fig. 5G–J). The up-regulation of PTGES in SkBr3 and MCF-7 cells treated with E2 and G-1 and cultured with conditioned medium from CAFs exposed to these ligands was not altered by increasing concentrations of the ER antagonist ICI up to 10 μM (data not shown). Collectively, these findings suggest that estrogenic GPER signalling generates a feedforward loop that couples IL1β induction in CAFs to IL1R1 expression by cancer cells, hence contributing to the functional cross-talk between the tumor microenvironment and breast cancer cells.

### GPER and IL1β/IL1R1 signalling cooperate in breast cancer cells

**Figure 6:**
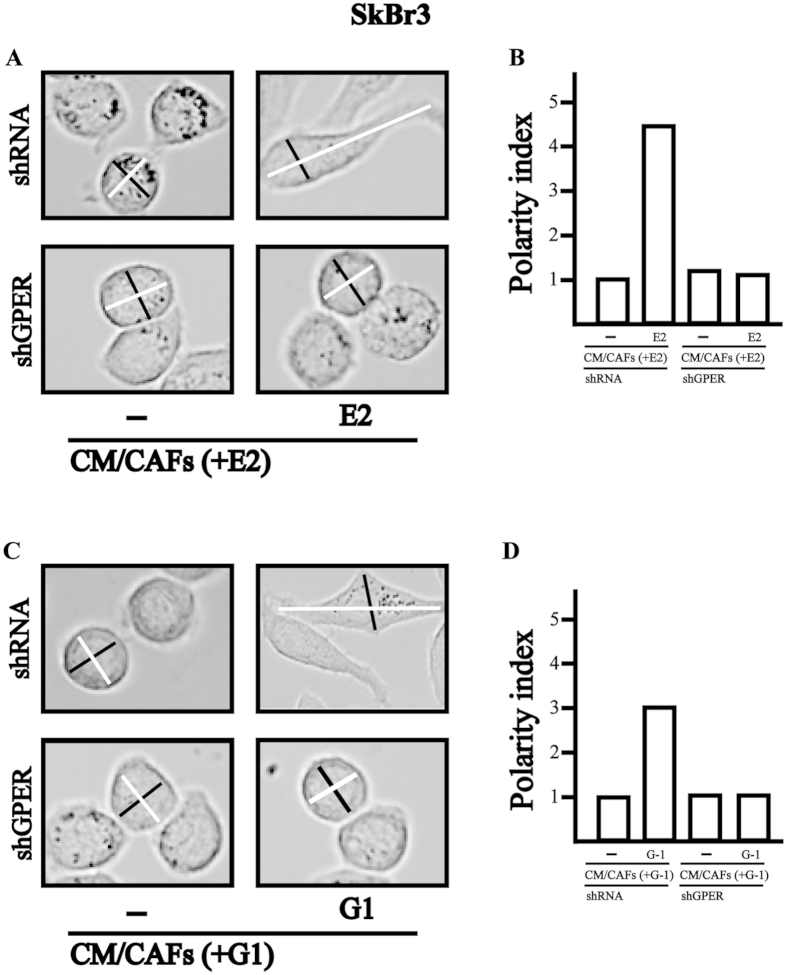
(**A**–**D**) SkBr3 cells were transfected for 24 h with shRNA or shGPER, treated for 8 h with vehicle (−), 10 nM E2 or 100 nM G-1 and then exposed for additional 8 h to conditioned medium collected from CAFs stimulated for 8 h with 10 nM E2 [CM/CAFs (+E2)] or 100 nM G-1 [CM/CAFs (+G-1)]. In panels (**A,C**) lines traced on cells were used to calculate the polarity index. White lines correspond to the migratory axis (MAx) and black lines to the transversal axis (TAx). In panels B and D, the polarity index (white migratory axis divided by black transversal axis) quantitatively defines the morphology of the migratory cell shown. Polarity Index =1.0 defines a polygonal shape, whereas a value >1.0 defines ranges of migratory shapes. Images shown are representative of 30 random fields obtained in three independent experiments.

**Figure 7:**
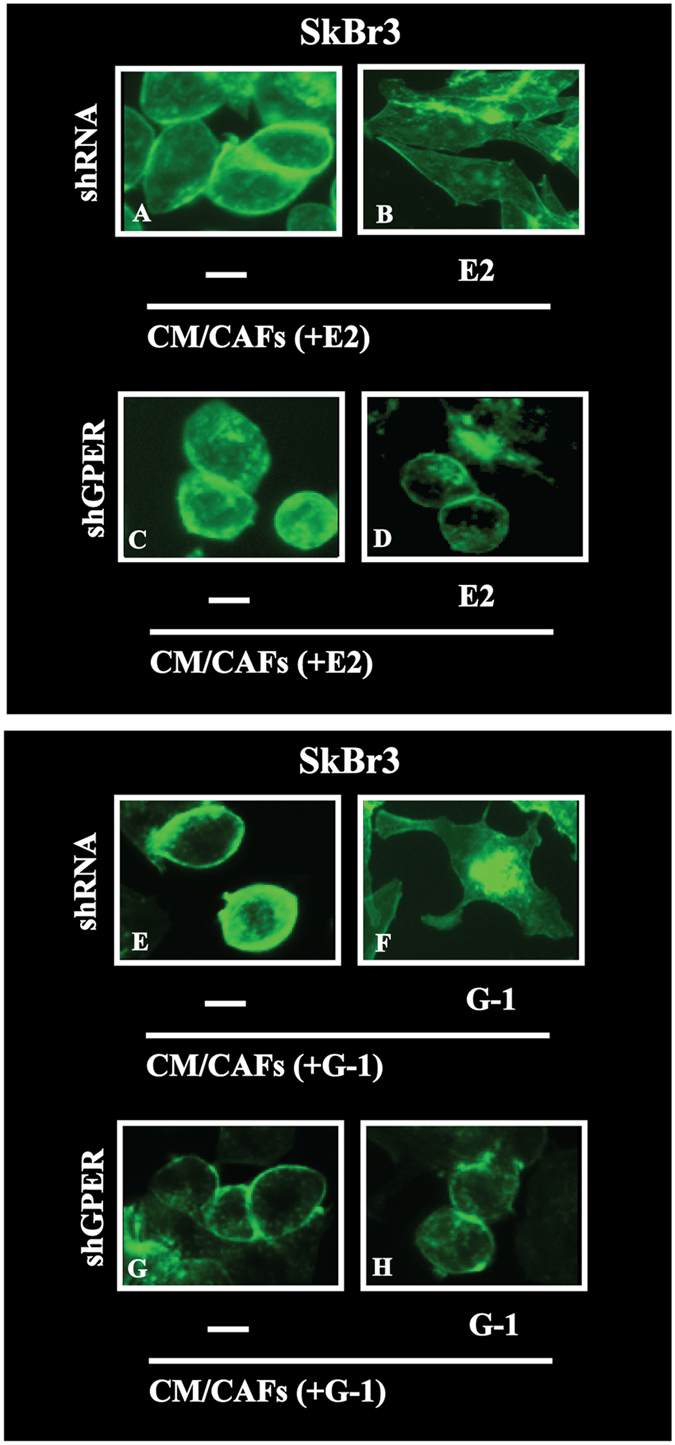
Actin cytoskeleton reorganization in SkBr3 cells transfected for 24 h with shRNA or shGPER and then treated for 8 h with vehicle (−) and 10 nM E2 (**A**–**D**) or vehicle (−) and 100 nM G-1 (**E**–**H**) before to be exposed for additional 8 h to conditioned medium collected from CAFs treated for 8 h with 10 nM E2 [CM/CAFs (+E2)] or 100 nM G-1 [CM/CAFs (+G-1)]. Cells were stained with Phalloidin-Fluorescent Conjugate (Santa Cruz Biotechnology) to visualize F-actin and analyzed using the Cytation 3 Cell Imaging Multimode Reader (BioTek, Winooski, VT). Images shown are representative of 30 random fields obtained in three independent experiments.

**Figure 8:**
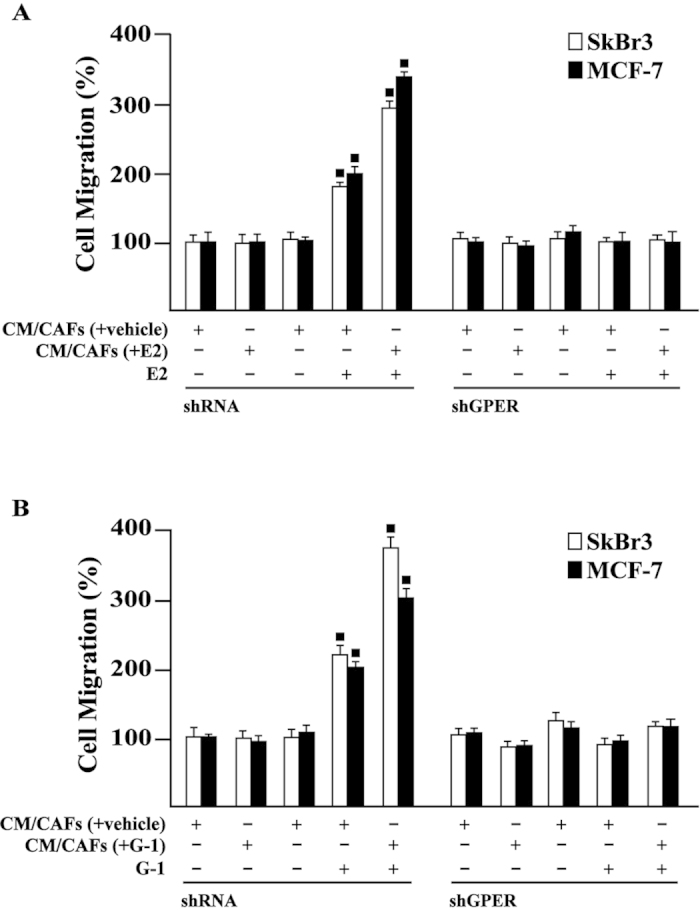
Migration assays performed by Boyden Chamber assay in SkBr3 and MCF-7 cells transfected for 24 h with shRNA or shGPER and then treated for 8 h with vehicle (−) and 10 nM E2 (**A**) or vehicle (−) and 100 nM G-1 (**B**) before to be exposed for additional 8 h to conditioned medium collected from CAFs treated for 8 h with vehicle, 10 nM E2 [CM/CAFs (+E2)] or 100 nM G-1 [CM/CAFs (+G-1)]. Each data point is the average ± SD of three independent experiments performed in triplicate. (◼) p < 0.05 for cells receiving treatments versus vehicle.

Upon IL1β stimulation, breast cancer cells acquire certain features of an invasive phenotype as the loss of cell-cell contact, the acquisition of a fibroblastoid cytoarchitecture and cell scattering^14^,^15^,^36^. Nicely recapitulating the abovementioned results, medium collected from E2 and G-1 treated CAFs induced a fibroblast-like phenotype (as evaluated by the polarity index) in SkBr3 cells transfected with a shRNA and exposed to E2 and G-1, but not in SkBr3 cells transfected with a shGPER (Fig. 6A–D). Findings similar to those obtained using medium collected from E2 and G-1 treated CAFs were elicited in SkBr3 cells exposed to E2 and G-1 before IL1β treatment (data not shown). Then, SkBr3 cells were fixed and stained with rhodamine-phalloidin to visualize the F-actin pattern. Conditioned medium from E2 and G-1 treated CAFs triggered the F-actin reorganization in SkBr3 cells transfected with a shRNA and exposed to E2 and G-1, but not in SkBr3 cells transfected with a shGPER (Fig. 7A–H). Results comparable to those obtained using medium collected from E2 and G-1 treated CAFs were elicited in SkBr3 cells exposed to E2 and G-1 before IL1β treatment (data not shown). The aforementioned findings were further supported by time-lapse video microscopy performed in MCF-7 cells treated with E2 and cultured with conditioned medium from CAFs exposed to E2 (videos 1–2). As previously shown^22^, E2 and G-1 stimulated the migration of SkBr3 and MCF-7 cells. This effect was further potentiated culturing cells with medium collected from E2 and G-1 treated CAFs, while the response was no longer observed in both cell types transfected with a shGPER (Fig. 8).

### GPER mediates IL1β up-regulation in CAFs derived from a cutaneous metastasis of breast cancer

The potential of GPER in regulating IL1β expression was also confirmed in CAFs derived from a cutaneous metastasis of an invasive mammary ductal carcinoma. In these cells lacking ERα and ERβ (data not shown) but expressing GPER mainly within the nuclear compartment (Supplementary Fig. 2A) as previously assessed in breast CAFs^25^, E2 and G-1 induced IL1β expression at both mRNA (Supplementary Fig. 2B,C) and protein levels (Supplementary Fig. 2D,E). Next, we found that the induction of IL1β upon exposure to E2 and G-1 occurs through GPER as its silencing abrogated the response (Supplementary Fig. 2F,G). Together, these results show that estrogenic GPER signalling may regulate IL1β expression also in CAFs derived from a breast cancer metastasis.

## Discussion

**Figure 9:**
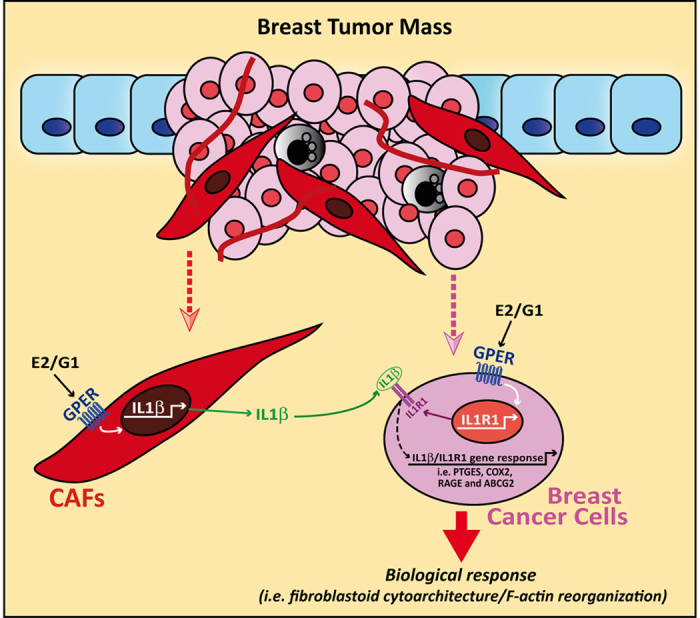
Schematic representation of ligand-activated GPER that generates a feedforward loop coupling IL1β induction by CAFs to IL1R1 expression by cancer cells, toward the induction of IL1β/IL1R1 target genes and biological responses as well as invasive features in breast cancer cells as fibroblastoid cytoarchitecture and F-actin reorganization.

In the present study we have shown that estrogenic GPER signalling triggers a feedforward loop which couples IL1β induction by CAFs to IL1R1 expression by cancer cells, toward the up-regulation of IL1β/IL1R1 target genes like PTGES, COX2, RAGE and ABCG2 and invasive features of breast cancer cells such as fibroblastoid cytoarchitecture and F-actin reorganization (see the schematic representation in Fig. 9). The aforementioned findings were confirmed, at least in part, in CAFs derived from a cutaneous metastasis of a breast malignancy. Altogether, these data provide novel insights into the potential of ligand-activated GPER to contribute to the functional interplay between cancer cells and the surrounding stroma toward the malignant progression.

Numerous factors are involved in the crosstalk between tumor cells and the associated stroma that influences disease initiation, progression and patient prognosis^37^. In particular, key components of the tumor microenvironment, namely CAFs, produce diverse secreted factors that sustain cancer aggressiveness targeting both cancer and stromal cells^38^. For instance, the pro-inflammatory cytokine CXCL12 produced by CAFs stimulate the proliferation and migration of tumor cells interacting with the cognate receptors expressed by cancer cells^39^. Other cytokines, chemokines and growth factors may also promote cancer-associated inflammation and metastasis inhibiting certain biological processes as the imbalance of oxidative stress, autophagy and angiogenesis^40^. Furthermore, CAFs can recruit immune cells responsible for the secretion of pro-inflammatory molecules, which contribute to tumor progression triggering immunosuppressive or ineffective host-antitumor responses^41^.

The cytokine IL1β is secreted by mononuclear phagocytes, keratinocytes, lymphocytes and cellular components of the tumor microenvironment^7^,^8^,^42^. IL1β, which is produced as an inactive precursor (pro-IL1b), is cleaved by the interleukin-converting enzyme and secreted in its mature form following tissue damage, infection and inflammation^6^. IL1β binding to and activating the cognate receptor IL1R1, stimulates diverse pathways like JNK, MAPK and NFkB, that lead to the production of inflammatory mediators and the regulation of biological responses like tissue vascularity, adipogenesis, lipid metabolism and inflammation^7^. As it concerns breast cancer, IL1β has been involved in the initiation, progression and invasiveness of this malignancy^43^,^44^,^45^. For instance, IL1β/IL1R1 system has been shown to up-regulate PTGES, which is a key enzyme involved in the production of COX2 and prostaglandin E_2_ that promote the motility of breast cancer cells^44^. Likewise, IL1β through IL1R1 stimulates the expression of genes linking inflammation and breast tumor, like RAGE and ABCG2^34^,^35^,^42^. Recapitulating these findings, we ascertained that IL1β/IL1R1 system mediates the transcription of the aforementioned genes induced by estrogenic GPER signalling in breast cancer cells. Moreover, our data may recall previous findings obtained either *in vitro* or *in vivo* showing that IL1β/IL1R1 axis plays a main role in the functional crosstalk between cancer cells and fibroblasts, leading to a pro-tumorigenic inflammatory phenotype^6^,^10^,^32^.

IL1β/IL1R1 activation promotes the motility of breast cancer cells, at least in part, through the stimulation of matrix metalloproteinases activity and morphological changes as fibroblast-like cellular phenotype characterized by a dynamic actin-rich lamellae and peripheral ruffles^14^,^46^. Nicely extending these data, in the present study medium collected from E2 and G-1 treated CAFs triggered the acquisition of a fibroblastoid cytoarchitecture and the reorganization of F-actin in breast cancer cells exposed to these GPER agonists. On the basis of these results, it could be assumed that estrogenic GPER signalling couples the expression of both IL1β in CAFs and IL1R1 in breast cancer cells, thus generating a feedforward IL1beta/IL1R1 response. Together, these findings suggest that ligand-activated GPER may play a role toward the inflammatory processes driving the progression of breast cancer. Moreover, the potential of GPER in contributing to the stimulatory effects elicited by estrogens has been previously shown using either cancer cells or CAFs^17^,^19^,^20^,^25^,^47^. For instance, GPER signalling activated the HIF-1α/VEGF signal transduction pathway leading to the stimulation of a main feature of tumor cells/stroma interaction such as hypoxia-induced angiogenesis^48^,^49^. To date, the multifaceted function of GPER in tumorigenesis is still a subject of deep debate. It should be mentioned that in previous studies GPER activation has been reported to inhibit cancer cell growth^50^. Further investigations have shown that high expression of GPER may be favorable for the survival of breast and ovarian cancer patients^51^,^52^,^53^. On the contrary, GPER mediated the expression of genes triggering tumor cell migration and proliferation both *in vitro* and *in vivo*^20^,^31^,^54^. In patients with endometrial and ovarian tumors, the expression of GPER was associated with aggressive features and lower survival rates^55^,^56^. Moreover, increased tumor size and metastasis of breast malignancies correlated with high levels of GPER expression^57^. GPER was also found increased and negatively correlated with relapse-free survival in patients treated with tamoxifen^53^. Next, the overexpression of GPER and its localization to the plasma membrane were suggested to be critical in breast cancer progression, whereas the absence of GPER in the plasma membrane predicted excellent long-term prognosis in breast cancer patients treated with tamoxifen^58^. Collectively, the results of these studies indicate that further investigations are needed in order to better understand the biological role exerted by GPER in different pathophysiological conditions. Here, we have demonstrated that GPER may integrate a feedforward IL1beta/IL1R1 response linking the tumor microenvironment with tumor cells toward the stimulation of breast cancer, as recapitulated in Fig. 9. The regulation of pro-inflammatory cytokines by estrogenic GPER signalling may be useful in order to set novel comprehensive therapeutic strategies targeting breast malignancy.

## Methods

### Reagents

17β-Estradiol (E2) was purchased from Sigma-Aldrich Srl (Milan, Italy). G-1 (1-[4-(-6-bromobenzol[1,3]diodo-5-yl)-3a,4,5,9b-tetrahidro3H5cyclopenta[c]quinolin-8yl]-ethanone) and G-15 (3aS,4R,9bR)-4-(6-bromo-1,3-benzodioxol-5-yl)-3a,4,5,9b-3H-cyclopenta[c]quinolone were obtained from Tocris Bioscience (Bristol, UK). Tyrphostin AG1478 (AG) was purchased from Biomol Research Laboratories, Inc (Milan, Italy). PD98059 (PD), bisindolylmaleimide I (GF109203X) (GF), LY294,002 (LY) and SB202190 (SB) were obtained from Calbiochem (Milan, Italy). H89 was purchased from Sigma-Aldrich Corp. (Milan, Italy). All the afore-mentioned compounds were dissolved in DMSO, except E2 which was solubilized in ethanol. Recombinant human IL1β was purchased from Thermo Fisher Scientific Inc. (Monza, Italy) and solubilized in PBS. IL-1 receptor antagonist (IL1R1a) human recombinant protein was purchased from Thermo Fisher Scientific Inc. (Monza, Italy) and solubilized in 20 mM TBS, pH 8, with 50% glycerol.

### Cell cultures

SkBr3 and MCF-7 breast cancer cells were obtained by ATCC (Manassas, VA, USA) and used <6 months after resuscitation. SkBr3 breast cancer cells were maintained in RPMI-1640 (Life Technologies, Milan, Italy) without phenol red, supplemented with 10% fetal bovine serum (FBS) and 100 μg/ml penicillin/streptomycin. MCF-7 breast cancer cells were cultured in DMEM (Dulbecco’s modified Eagle’s medium) (Life Technologies, Milan, Italy) with phenol red, supplemented with 10% FBS and 100 μg/ml penicillin/streptomycin. CAFs obtained from breast malignancies were characterized and maintained as we previously described^59^. CAFs were extracted from six invasive mammary ductal carcinomas obtained from mastectomies. In each patient, a second population of fibroblasts was isolated from a noncancerous breast tissue at least 2 cm from the outer tumor margin. Metastasis-derived CAFs were obtained from biopsy of cutaneous metastasis in a patient with a primary invasive mammary ductal carcinoma, who previously had undergone surgery. Briefly, specimens were cut into smaller pieces (1–2 mm diameter), placed in digestion solution (400 IU collagenase, 100 IU hyaluronidase, and 10% serum, containing antibiotic and antimycotic solution) and incubated overnight at 37 °C. The cells were then separated by differential centrifugation at 90 × g for 2 min. Supernatant containing fibroblasts was centrifuged at 485 × g for 8 min; the pellet obtained was suspended in fibroblasts growth medium (Medium 199 and Ham’s F12 mixed 1:1 and supplemented with 10% FBS) and cultured at 37 °C in 5% CO2. Primary cells cultures of metastasis-derived fibroblasts were characterized by immunofluorescence. Briefly cells were incubated with human anti-vimentin (V9) and human anti-cytokeratin 14 (LL001), both from Santa Cruz Biotechnology (DBA, Milan, Italy). To characterize fibroblasts activation, we used anti-fibroblast activated protein α (FAPα) antibody (H-56; Santa Cruz Biotechnology, DBA, Milan, Italy) (data not shown). CAFs and metastasis-derived CAFs were maintained in Medium 199 and Ham’s F12 (mixed 1:1) supplemented with 10% FBS and 100 μg/ml penicillin/streptomycin and cultured at 37 °C in 5% CO2. Signed informed consent from all the patients was obtained and all samples were collected, identified and used in accordance with approval by the Institutional Ethical Committee Board (Regional Hospital, Cosenza, Italy). All cell lines were grown in a 37 °C incubator with 5% CO2. All cell lines to be processed for immunoblot and RT-PCR assays were switched to medium without serum and phenol red the day before treatments.

### Gene expression studies

Total RNA was extracted and cDNA was synthesized by reverse transcription as previously described^60^. The expression of selected genes was quantified by real-time PCR using platform Quant Studio7 Flex Real-Time PCR System (Life Technologies). Gene-specific primers were designed using Primer Express version 2.0 software (Applied Biosystems). For IL1β, IL1R1, PTGES, RAGE, ABCG2, COX2 and the ribosomal protein 18S, which was used as a control gene to obtain normalized values, the primers were: 5′-ACGATGCACCTGTACGATCA-3′ (IL1β forward) and 5′-TGCTTGAGAGGTGCTGATGT-3′ (IL1 β reverse); 5′-AACAGACAGGGCCTAGCTTT-3′ (IL1R1 forward) and 5′-TCAAAGGAAGTTCACGGGGA-3′ (IL1R1 reverse); 5′-CATCAACTTTCCGGGGGTGA-3′ (ABCG2 forward) and 5′-ACCAACAGACCATCATAAACACA-3′ (ABCG2 reverse); 5′-CCCTTCTGCCTGACACCTTT-3′ (COX2 forward) and 5′-GCCTGCTCTGGTCAATGGAA-3′ (COX2 reverse); 5′-CCCAAGGTTTGAGTCCCTCC-3′ (PTGES forward) and 5′- CACATCTCAGGTCACGGGTC-3′ (PTGES reverse); 5′-CGTAAAGATGGGGGCTGGAG-3′ (RAGE forward) and 5′-ACCTTCCAAGCTTCTGTCCG-3′ (RAGE reverse); 5′-GGCGTCCCCCAACTTCTTA-3′ (18S forward) and 5′-GGGCATCACAGACCTGTTATT-3′ (18S reverse). Assays were performed in triplicate and the results were normalized for 18S expression and then calculated as fold induction of RNA expression.

### Western Blot Analysis

Cells were grown in 10-cm dishes, exposed to treatments and then lysed in 500 μL of 50 mmol/L NaCl, 1.5 mmol/L MgCl2, 1 mmol/L EGTA, 10% glycerol, 1% Triton X-100, 1% sodium dodecyl sulfate (SDS), and a mixture of protease inhibitors containing 1 mmol/L aprotinin, 20 mmol/L phenylmethylsulfonyl fluoride and 200 mmol/L sodium orthovanadate. Protein concentration was determined using Bradford reagent according to the manufacturer’s recommendations (Sigma-Aldrich, Milan, Italy). Equal amounts of whole protein extract were resolved on a 10% SDS-polyacrylamide gel, transferred to a nitrocellulose membrane (Amersham Biosciences, GE Healthcare, Milan, Italy), probed overnight at 4 °C with antibodies against IL1β (R&D Systems, Inc. Celbio, Milan, Italy), IL1R1 (OriGene Technologies, TEMA ricerca srl, Bologna, Italy), GPER (N-15), PGE synthase (S-16) and β-actin (C-2) all purchased from Santa Cruz Biotechnology, DBA, Milan, Italy). Proteins were detected by horseradish peroxidase-linked secondary antibodies (Santa Cruz Biotechnology, DBA) and revealed using the ECL System (GE Healthcare).

### Gene Silencing Experiments

Cells were plated onto 10-cm dishes and transfected using X-treme GENE 9 DNA Transfection Reagent (Roche Diagnostics, Milan, Italy) for 24 hours before treatments with a control shRNA or a shRNA specific for GPER (shGPER). The silencing of GPER expression was obtained by the construct which we have previously described and used^60^.

### Enzyme-Linked Immunosorbent Assay

The concentrations of IL-1β in supernatants from E2 and G-1 treated CAFs were evaluated by enzyme-linked immunosorbent assay (ELISA) according to the manufacturers’ protocols (Thermo Fisher Scientific Inc.).

### Conditioned medium

CAFs were cultured in regular growth medium, switched to medium without serum and phenol red for 24 h and then treated for 8 h with E2 or G-1. Thereafter, the supernatants were collected and used as conditioned medium in SkBr3 and MCF-7 cells.

### Polarization assay

SkBr3 cells were serum deprived and transfected for 24 h with a control shRNA or shGPER using X-tremeGene9 reagent (Roche Molecular Biochemical), as recommended by the manufacturer, and then treated for 8 h with vehicle (–), E2 (10 nM) or G-1 (100 nM) before to be exposed for additional 8 h to conditioned medium from CAFs treated for 8 h with E2 or G-1. Then cells were fixed in 4% paraformaldehyde. After washed with PBS, images were acquired using the Cytation 3 Cell Imaging Multimode Reader (BioTek, Winooski, VT). For each individual cell, the polarity index (PI) was calculated dividing the length of the long migration-defined axis by the perpendicular axis passing by the centroid of the cell^36^.

### F-actin staining

Cells were transfected, treated and fixed as indicated above. Thereafter, cells were washed with PBS, incubated with Phalloidin-Fluorescent Conjugate (Santa Cruz Biotechnology, DBA) to visualize F-actin and analyzed using the Cytation 3 Cell Imaging Multimode Reader (BioTek, Winooski, VT).

### Migration assay

Migration assays were performed in triplicate using Boyden chambers (Costar Transwell, 8 mm polycarbonate membrane, Sigma Aldrich, Milan, Italy). SkBr3 and MCF-7 cells were transfected for 24 hours with shRNA or shGPER in regular growth medium. Thereafter, cells were treated with ligands for 8 h, then trypsinized and seeded in the upper chambers. Conditioned medium from CAFs treated with ligands was added in the bottom wells for 8 hours, then cells on the bottom side of the membrane were fixed and counted.

### Immunofluorescence microscopy

Metastasis-derived CAFs were seeded in Lab-Tek II chamber slides at a density of 1 × 10^5^ per well and incubated for 24 h in the corresponding maintenance media. For immunofluorescence staining, cells were transfected for 24 h, fixed in 4% paraformaldehyde, permeabilized with 0.1% TWEEN three times for 5min and then were blocked for 30 min at room temperature with PBS containing 10% normal donkey serum (Santa Cruz Biotechnology, DBA, Milan, Italy), 0.1% Triton X-100, and 0.05% TWEEN. Thereafter, cells were incubated overnight at 4 °C with a primary antibody against GPER (K-19) (1:100 purchased from Santa Cruz Santa Cruz Biotechnology, DBA, Milan, Italy) in PBS containing 0.05% TWEEN. After incubation, the slides were extensively washed with PBS and incubated with donkey anti-rabbit IgG-FITC (1:100, from Santa Cruz Biotechnology, DBA, Milan, Italy) and 4′,6-Diamidino-2-phenylindole dihydrochloride (DAPI) (1:1000, Sigma-Aldrich, Milan, Italy). The slides were imaged on the Cytation 3 Cell Imaging Multimode reader (BioTek, Winooski, VT) and analysed using the software Gen5 (BioTek, Winooski, VT).

### Time-lapse microscopy

MCF-7 cells (1 × 10^5^) were seeded for 24 hours in 6-well plates in regular growth medium and cultured thereafter in medium without serum in the presence of E2 for 8 hours. Then, cells were cultured in conditioned medium from CAFs exposed to E2 for 8 hours. Cells were maintained at routine incubation settings (37 °C, 5% CO_2_) using Cytation™3 Cell Imaging Multi-Mode Reader (Biotek, Winooski, VT). In order to evaluate the fibroblastoid cytoarchitecture and cell scattering, the images were recorded in 10 min intervals for 8 hours culturing MCF-7 cells in conditioned medium from CAFs (software Gen5, BioTek, Winooski, VT). The images that were processed as a movie using the software Adobe Creative Cloud Premier Pro CC. Frames, are displayed at a rate of 10 frames s-1.

### Statistical analysis

Statistical analysis was performed using ANOVA followed by Newman-Keuls’ testing to determine differences in means. p < 0.05 was considered statistically significant.

## Additional Information

**How to cite this article**: De Marco, P. *et al.* GPER signalling in both cancer-associated fibroblasts and breast cancer cells mediates a feedforward IL1β/IL1R1 response. *Sci. Rep.* **6**, 24354; doi: 10.1038/srep24354 (2016).

## Supplementary Material

Supplementary Information

Supplementary video 1

Supplementary video 2
