# Supplementary material for: GPER signalling in both cancer-associated fibroblasts and breast cancer cells mediates a feedforward IL1β/IL1R1 response
Source: Sci Rep. 2016 Apr 13;6:24354. doi: 10.1038/srep24354 (PMC4829876; doi:10.1038/srep24354)
Supplement: Supplementary Information [file srep24354-s1.pdf]

# GPER signalling in both cancer-associated fibroblasts and breast cancer cells mediates a feedforward IL1β/IL1R1 response

Paola De Marco, Rosamaria Lappano, Ernestina Marianna De Francesco, Francesca Cirillo, Marco Pupo, Silvia Avino, Adele Vivacqua, Sergio Abonante, Didier Picard & Marcello Maggiolini

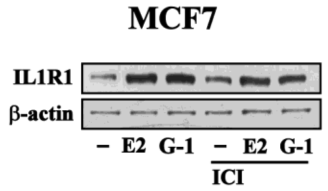


**Supplementary Fig.1**

## Metastasis-derived CAFs

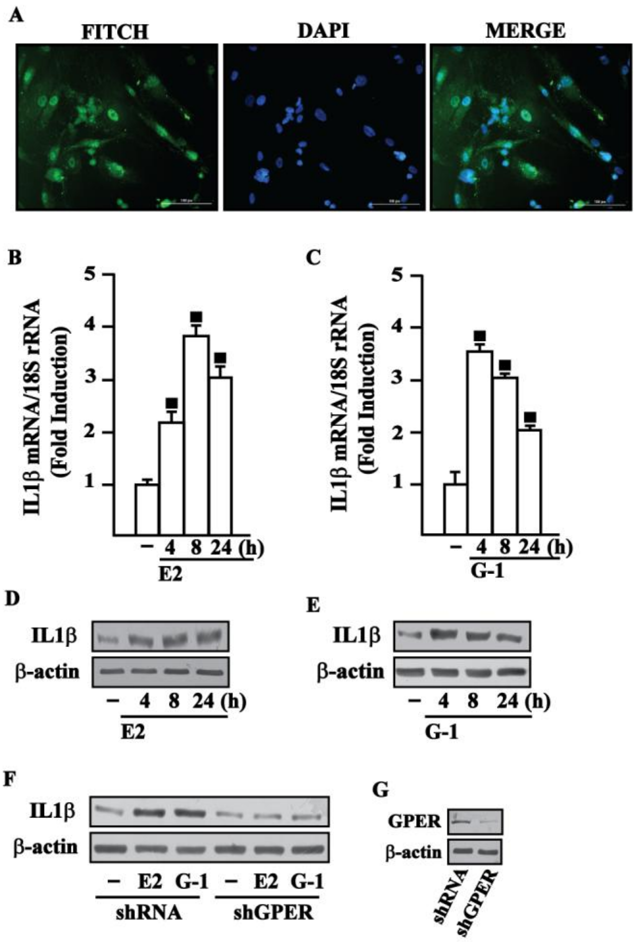


**Supplementary Fig.2**

## Supplementary Figure Legends

**Supplementary Figure 1.** IL1R1 protein expression upon MCF-7 cells exposure for 8 h to 10 nM E2 and 100 nM G-1 alone and in the presence of 10 μM ER antagonist ICI 182 780 (ICI). β-actin serves as a loading control. Results shown are representative of two independent experiments.

**Supplementary Figure 2.** IL1β expression is up-regulated by E2 and G-1 through GPER in CAFs derived from cutaneous metastasis of a breast cancer patient. (A) GPER localization in metastasis-derived CAFs. Representative fluorescence images of cells immunostained with anti-GPER antibody (green). Nuclei were stained by DAPI (blue). Scale bars, 100 μm. Each experiment shown is representative of 30 random fields observed each in two independent experiments. (B) 10 nM E2 and (C) 100 nM G-1 induce the mRNA expression of IL1β, as evaluated by real-time PCR. Data obtained in three independent experiments performed in triplicate were normalized to 18S expression and shown as fold changes of IL1β expression upon E2 and G-1 treatments respect to cells exposed to vehicle (-). (■) $p<0.05$ for cells receiving treatments versus vehicle. (D) 10 nM E2 and (E) 100 nM G-1 up-regulate IL1β protein expression, as indicated. (F) The up-regulation of IL1β protein levels observed upon a 8 h exposure to 10 nM E2 and 100 nM G-1 in metastasis-derived CAFs is no longer evident transfecting cells for 24 h with shGPER before treatments. (G) Efficacy of GPER silencing. β-actin serves as a loading control. Results shown are representative of at least two independent experiments.

**Videos 1-2.** Time-lapse video microscopy of live MCF-7 cells treated for 8 h with 10 nM E2 and then exposed for additional 8 h to conditioned medium collected from CAFs (CM/CAFs) which were treated for 8 h with vehicle [CM/CAFs (+vehicle)] and 10 nM E2 [CM/CAFs (+E2)]. Images were acquired every 10 minutes for 8 h using Cytation 3 Cell Imaging Multimode Reader and the software Gen5 (BioTek, Winooski, VT). Frames collected every 10 minutes are displayed at a rate of 10 frames s-1.
